# Trends in infants born at low birthweight and disparities by maternal race and education from 2003 to 2018 in the United States

**DOI:** 10.1186/s12889-021-11185-x

**Published:** 2021-06-10

**Authors:** Elizabeth A. Pollock, Keith P. Gennuso, Marjory L. Givens, David Kindig

**Affiliations:** University of Wisconsin Population Health Institute, University of Wisconsin Madison School of Medicine and Public Health, 610 N Walnut Street, Madison, WI 53726 USA

**Keywords:** United States, Low birthweight, Health disparities, Race, Education, Trend analysis

## Abstract

**Background:**

Understanding current levels, as well as past and future trends, of the percentage of infants born at low birthweight (LBW) in the United States is imperative to improving the health of our nation. The purpose of this study, therefore, was to examine recent trends in percentage of LBW, both overall and by maternal race and education subgroups. Studying disparities in percentage of LBW by these subgroups can help to further understand the health needs of the population and can inform policies that can close race and class disparities in poor birth outcomes.

**Methods:**

Trends of percentage of LBW in the U.S. from 2003 to 2018, both overall and by race/ethnicity, and from 2007 to 2018 by education and race by education subgroups were analyzed using CDC WONDER Natality data. Disparities were analyzed using between group variance methods.

**Results:**

Percentage of LBW experienced a significant worsening in the most recent 5 years of data, negating nearly a decade of prior improvement. Stark differences were observed by race/ethnicity and by education, with all subgroups experiencing increasing rates in recent years. Disparities also worsened over the course of study. Most notably, all disparities increased significantly from 2014 to 2018, with annual changes near 2–5%.

**Conclusions:**

Recent reversals in progress in percentage of LBW, as well as increasing disparities particularly by race, are troubling. Future study is needed to continue monitoring these trends and analyzing these issues at additional levels. Targets must be set and solutions must be tailored to population subgroups to effectively make progress towards equitable birth outcomes and maternal health.

**Supplementary Information:**

The online version contains supplementary material available at 10.1186/s12889-021-11185-x.

## Introduction

The percentage of infants born at low birthweight (LBW), or the percentage of live births where the infant weighed less than 2500 g, is an important population health outcome measure, reflecting both maternal health and infant health. LBW can serve as a reference for both current and future health of society’s youngest age group, serving as a predictor of premature mortality risk as well as morbidity over the child’s life course [1–3]. Children born at LBW face myriad health issues over the life course, including greater risk of developmental and growth problems, cardiovascular disease, and respiratory conditions. They also have higher rates of cognitive problems such as cerebral palsy, and visual, auditory, and intellectual impairments [4–7]. As a maternal health outcome, birthing an infant born at LBW indicates a mother’s exposure to health risks in multiple categories of health factors, including her health behaviors, access to health care, the social and economic environment the mother inhabits, and environmental risks to which she is exposed. LBW as an outcome is also disproportionately prevalent among certain population groups, particularly those that have experienced inequities, or unfair disparities, like mothers with lower social and economic status and mothers who are Black, due in part to unequal opportunity, differential access to quality health care, and chronic stress related to economic or social adversity such as discrimination and racism [8–10].

For these reasons and numerous more, understanding current levels, as well as past and future trends, of percentage of LBW in the United States is imperative to improving the health of our nation. As a measure, percentage of LBW has its limitations, including relying on a relatively arbitrary weight cut-off and resulting from a multitude of upstream factors, some of which are expressly negative as mentioned above and some of which may be positive such as improvements in treating infertility or monitoring at-risk pregnancies. Yet, it remains a useful marker of population health. While there are groups that track and analyze trends in percentage of LBW in the United States [10–13], there is a need for deeper study and exposure of this metric, particularly in clearly defining and testing trend periods of and disparities in percentage of LBW for multiple identities of population subgroups. The purpose of this study, therefore, was to examine recent trends in percentage of LBW, both overall and by maternal race/ethnicity and education subgroups. Studying disparities in percentage of LBW by these subgroups can help to further understand the health needs of the population and can inform policies that can close race and class disparities in poor birth outcomes.

## Methods

Data on percentage of LBW and maternal characteristics were collected from the CDC WONDER Natality files using single-year estimates, from 2003 to 2018 [14]. Natality information is collected by the CDC from birth certificates, reporting counts of live births occurring in the U.S. along with demographic information. Births for which the infant birthweight was unknown or not stated on the certificate were excluded from the sample. Birth data were restricted to singleton births in order to avoid biasing the estimates from multiples who tend to be smaller. Percentage of LBW data were collected for the entire U.S. population, as well as for subsets by maternal race, education, and the combination of race by education. Maternal race/ethnicity of the infant was categorized into mutually exclusive groups of non-Hispanic American Indian and Alaska Native, non-Hispanic Asian, non-Hispanic Black, non-Hispanic White, and Hispanic, hereon referred to as AIAN, Asian, Black, White, and Hispanic, respectively. Mothers identifying as more than one race were not captured under these definitions, and mothers who identified as Hispanic ethnicity were assigned to Hispanic, regardless of the race reported. Infants were grouped into four maternal education categories: less than high school education, high school graduate (or GED), some college credit but no degree, and college degree or higher, hereon referred to as less than high school, high school, some college, and college, respectively. Lastly, the race by education domain was broken down into 20 distinct groups, using combinations of the 5 race/ethnicity and 4 education categories. The time period for analysis for race began in 2003 due to changes in racial/ethnic data collection standards, and the time period for analysis including education began in 2007 due to a lack of data availability and inconsistency of educational definitions in birth certificates prior to 2007.

Trends in percentage of LBW were examined with joinpoint, or segmented, regression modeling methods using the National Cancer Institute’s Joinpoint Regression Program version 4.6.0.0 [15]. This is a non-linear form of analysis to examine trends in data that allows for the identification of time points, otherwise known as “joinpoints” or “knots”, where the trend significantly changes [16, 17]. The joinpoints and the annual percentage change (APC) of trend lines between them were determined based on logarithmically transformed percentage of LBWs and their standard errors. A maximum number of two joinpoints were allowed, based on the available years of data.

Since parity is inherently a comparative concept, choice of disparity metric is important, subjective, and reflects normative values and judgments [18]. In order to conceptualize variation in the population and to not label any groups as most favorable, normal, or ideal, the population average was chosen for comparisons, as well as the use of population weighting to account for the population sizes of subgroups. Accordingly, analyses of disparities by maternal race, education, and their combination were conducted using between group variance (BGV) methods. BGV calculates the variance of grouped data by squaring the differences in group rates from the population average and weights by their population sizes, with a higher value indicating higher disparity, making the metric a useful indicator of absolute disparity for unordered group data [19]. Trends in the BGVs were then examined using the above joinpoint methods.

Secondary analyses examined trends for very low birthweight (VLBW), or the percentage of live births where the infant weighed less than 1500 g, to understand if the LBW cut-off of 2500 g strongly affected results. Multiple births, or babies born as twins, triplets, quadruplets, quintuplets, or higher, were also analyzed to understand outcomes and trends in this seldom-studied group. Additional analyses examined trends by advanced maternal age, defined by births from mothers aged 35 years and older, to understand if findings differ for mothers who choose to have children later in life or are more likely to use in vitro fertilization, both of which are associated with smaller infants [20, 21]. All analyses were performed in Microsoft Excel and Joinpoint Regression Program in 2020 and 2021. Institutional review board approval was not required because no human participants were included in this study.

## Results

**Fig. 1:**
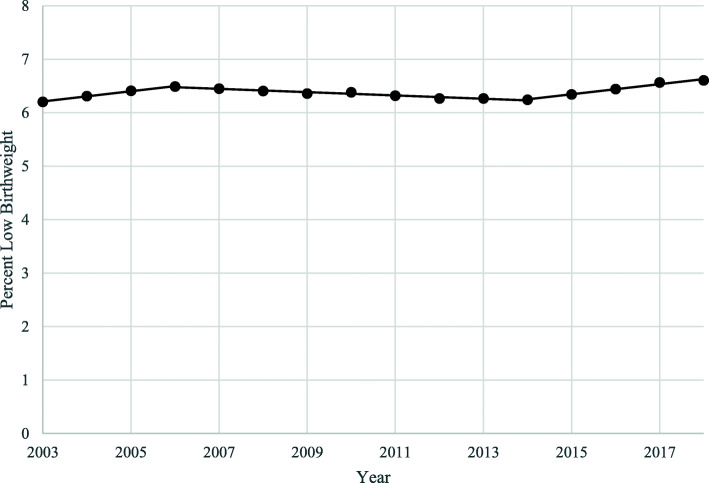
Trends in LBW, United States, 2003–2018. LBW = Low Birthweight

Table 1 shows the number of LBW births and the percentage of LBW for the overall U.S. and all subgroups during the baseline year and 2018. Between 2003 and 2018, percentage of LBW in the entire U.S. increased from 6.2 to 6.6%, but the overall trend for the time period was nearly flat. Figure 1 displays the change in overall percentage of LBW between 2003 and 2018 across the entire U.S. for all births and identifies three distinct trend periods occurring over this time. Joinpoint analysis revealed that from 2003 to 2006, percentage of LBW was significantly increasing in the U.S., at a rate of 1.4% per year. From 2006 to 2014, percentage of LBW then significantly decreased with an APC of − 0.5. Finally, from 2014 to 2018, there was another reversal in the trend, significantly increasing with a rate of 1.6% per year, which negated nearly a decade of improvement. Table 2 provides the joinpoint trend data for all groups, including the trend period years and accompanying APCs.

**Table 1:** LBW overall and by maternal race, education, and their combination, United States, baseline and 2018

	Baseline LBW births^a^	Baseline Percent LBW^a^	2018 LBW Births	2018 Percent LBW
U.S. Overall	245,104	6.2%	241,875	6.6%
Race				
AIAN	2330	6.2%	2229	6.8%
Asian	13,056	6.3%	17,907	7.0%
Black	64,233	11.6%	64,667	11.7%
Hispanic	49,480	5.6%	53,562	6.2%
White	114,183	5.1%	101,102	5.3%
Education^b^				
Less than HS	37,118	7.3%	39,162	8.5%
HS	41,440	7.0%	73,589	7.8%
Some College	26,195	6.2%	50,369	6.9%
College	29,984	4.6%	74,687	5.0%
Race by Education				
AIAN, Less than HS	262	7.4%	543	8.4%
AIAN, HS	218	5.8%	823	7.0%
AIAN, Some College	148	5.1%	547	6.5%
AIAN, College	75	5.1%	293	5.0%
Asian, Less than HS	641	7.3%	1401	7.7%
Asian, HS	1336	6.5%	2599	7.6%
Asian, Some College	1221	6.9%	2108	7.4%
Asian, College	4564	6.1%	11,563	6.7%
Black, Less than HS	7680	13.2%	10,095	13.8%
Black, HS	10,369	12.1%	24,439	12.5%
Black, Some College	6650	10.8%	15,996	11.3%
Black, College	3742	9.2%	13,380	9.6%
Hispanic, Less than HS	17,521	5.8%	15,057	6.7%
Hispanic, HS	11,262	5.7%	17,621	6.3%
Hispanic, Some College	5616	5.9%	10,514	6.2%
Hispanic, College	3751	4.9%	9613	5.4%
White, Less than HS	10,833	8.3%	11,901	8.7%
White, HS	18,067	6.4%	27,808	6.7%
White, Some College	12,466	5.2%	21,011	5.6%
White, College	17,754	3.9%	39,499	4.0%

*LBW* Low Birthweight, *AIAN* American Indian and Alaska Native, *HS* High School

^a^ Baseline values are for 2003 for Overall and Race and are for 2007 for Education and Race by Education

^b^ Births for education fluctuated because there were nearly two million births for which education was missing or excluded on the birth certificate in 2007 and less than 50,000 in 2018

**Table 2:** Trends in LBW overall and by maternal race, education, and their combination, United States, 2003–2018

	Trend period 1 Years	Trend period 1 APC	Trend period 2 Years	Trend period 2 APC	Trend period 3 Years	Trend period 3 APC
U.S. Overall	2003–2006	1.4*	2006–2014	−0.5*	2014–2018	1.6*
Race						
AIAN	2003–2018	0.6*				
Asian	2003–2014	0.2	2014–2018	1.6*		
Black	2003–2005	1.7*	2005–2014	−1.0*	2014–2018	1.6*
Hispanic	2003–2006	1.1*	2006–2014	0.1*	2014–2018	1.8*
White	2003–2006	1.5*	2006–2013	−0.8*	2013–2018	0.8*
Education						
Less than HS	2007–2018	1.3*				
HS	2007–2014	0.7*	2014–2018	1.8*		
Some College	2007–2015	0.7*	2015–2018	1.9*		
College	2017–2014	0.0	2014–2018	1.9*		
Race by Education						
AIAN, Less than HS	2007–2018	1.5*				
AIAN, HS	2007–2018	1.7*				
AIAN, Some College	2007–2018	1.7*				
AIAN, College	2007–2018	−0.5				
Asian, Less than HS	2007–2018	1.3*				
Asian, HS	2007–2018	1.0*				
Asian, Some College	2007–2018	0.4†				
Asian, College	2007–2014	−0.1	2014–2018	2.4*		
Black, Less than HS	2007–2013	−0.5†	2013–2018	1.4*		
Black, HS	2007–2013	−0.7†	2013–2018	1.9*		
Black, Some College	2007–2013	−0.5*	2013–2018	1.4*		
Black, College	2007–2013	−0.6†	2013–2018	1.1*		
Hispanic, Less than HS	2007–2014	0.7*	2014–2018	2.5*		
Hispanic, HS	2007–2014	0.3	2014–2018	1.8*		
Hispanic, Some College	2007–2014	−0.1	2014–2018	1.6*		
Hispanic, College	2007–2018	0.7*				
White, Less than HS	2007–2018	0.6*				
White, HS	2007–2018	0.7*				
White, Some College	2007–2018	0.9*				
White, College	2007–2013	−0.6*	2013–2018	1.1*		
Disparities						
BGV Race	2003–2006	1.4	2006–2014	−2.8*	2014–2018	4.9*
BGV Education	2007–2009	1.6	2009–2012	8.0*	2012–2018	2.7*
BGV Race by Education	2007–2018	1.7*				

Periods refer to before and after an inflection point in the overall trend, detected by joinpoint regression analysis. Inflection points were determined independently by group and are not intended to warrant comparisons of trend periods between groups

*LBW* Low Birthweight, *AIAN* American Indian and Alaska Native, *HS* High School, *BGV* Between Group Variance, *APC* Annual Percentage Change, determined using joinpoint regression

* *p*-value < 0.05

† *p*-value < 0.1

**Fig. 2:**
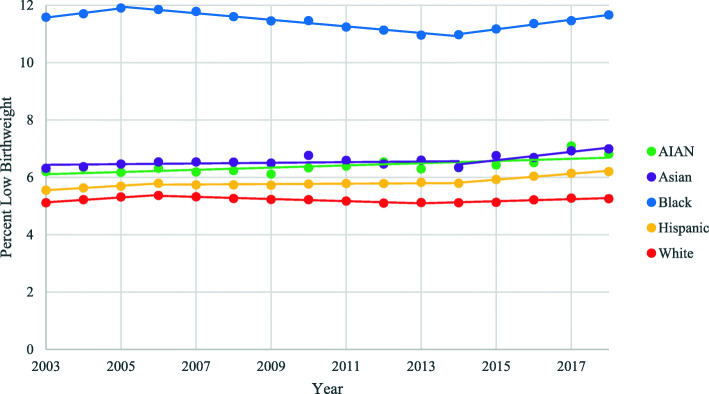
Trends in LBW by Maternal Race, United States, 2003–2018. LBW = Low Birthweight. AIAN = American Indian and Alaska Native

Looking at trends by maternal race (Fig. 2), percentage of LBW to White and Black mothers followed similar trends to overall percentage of LBW in the U.S. There were three distinct, statistically significant trend periods for percentage of LBW among both White and Black mothers during this time. From 2003 to 2005, percentage of LBW among Black mothers increased with an APC of 1.7, decreased from 2005 to 2014 with an APC of − 1.0, and increased again from 2014 to 2018 with an APC of 1.6. Percentage of LBW among White mothers increased from 2003 to 2006 with an APC of 1.5, decreased from 2006 to 2013 at a rate of − 0.8, and increased from 2013 to 2018 at a rate of 0.8. Percentage of LBW among AIAN, Asian, and Hispanic mothers, on the other hand, did not experience the significant decrease in the middle of the time period, but rather all saw increasing trends over the time period, particularly over the most recent 5 years, with APCs of 0.6, 1.6, and 1.8, respectively. It is also important to note that percentage of LBW among Black mothers was more than twice as high as White mothers (11.7 and 5.3% in 2018, respectively) with AIAN, Asian, and Hispanic rates falling in the middle (6.8, 7.0%, and 6.2 percentage of LBW in 2018, respectively).

**Fig. 3:**
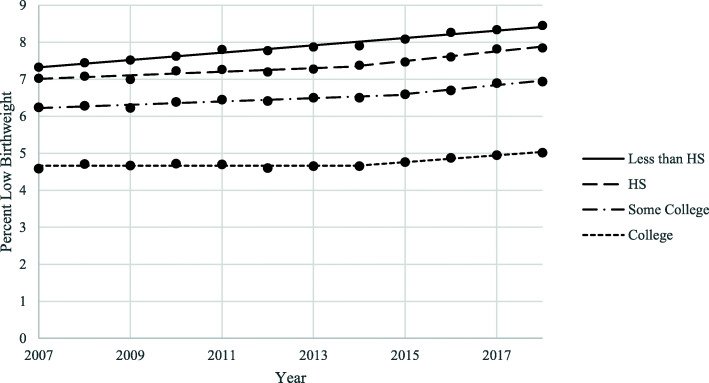
Trends in LBW by Maternal Education, United States, 2007–2018. LBW = Low Birthweight. HS = High School

Examining trends by maternal education (Fig. 3), percentage of LBW among mothers with all education levels experienced increasing trends from 2007 to 2018, with 2018 percentage of LBW values of 8.5% for less than high school, 7.8% for high school, 6.9% for some college, and 5.0% for college; showing a gradient improvement in rates as education level grows. Percentage of LBW among mothers with less than high school education increased steadily over the whole time period with a statistically significant APC of 1.3. Percentage of LBW among mothers in the high school graduate group increased significantly from 2007 to 2014 with an APC of 0.7 and then again from 2014 to 2018 at 1.8% per year. Percentage of LBW among mothers with some college education increased significantly from 2007 to 2015 with an APC of 0.7 and at a higher rate from 2015 to 2018 with an APC of 1.9. Finally, percentage of LBW among mothers with a college education or more stayed flat from 2007 to 2014 and increased significantly from 2014 to 2018 with an APC of 1.9.

**Fig. 4:**
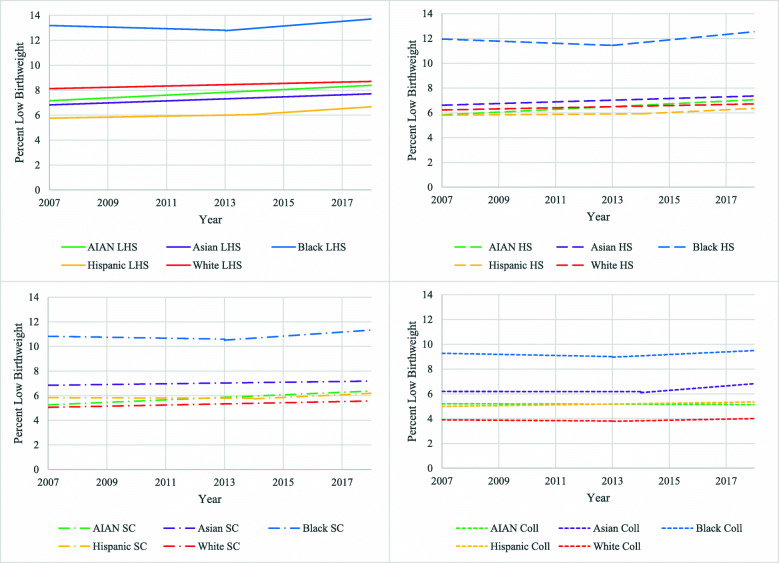
Trends in LBW by Maternal Race and Education Combined, United States, 2007–2018. LBW = Low Birthweight. AIAN = American Indian and Alaska Native. LHS = Less than High School. HS = High School. SC = Some College. Coll = College

The data were then further broken down into twenty mutually exclusive, race by education subgroups (Fig. 4). Percentage of LBW for Black mothers among all education categories was higher than any of the other 16 race by education subcategories throughout the entire time period, ranging between 9.6 and 13.8%. Percentage of LBW was lowest for White mothers with college or higher education at 4.0%, while percentage of LBW for White mothers with less than high school education at 8.7% was higher than all other subcategories apart from the Black subcategories. All race by education subgroups saw increasing trends over the time period, with the sole exception of percentage of LBW for AIAN mothers with a college degree or higher, which decreased from 2007 to 2018 from 5.1 to 5.0% with an APC of − 0.5. Hispanic mothers with less than high school education saw the largest increase over the most recent period, rising from 6.0% in 2014 to 6.7% in 2018 with a statistically significant APC of 2.5. In terms of absolute numbers, however, the greatest number of infants born at low birthweight were to the highly educated, White mothers at more than 39,000 despite this group having the lowest rate. All trends can be seen in Table 2 and Fig. 4.

**Fig. 5:**
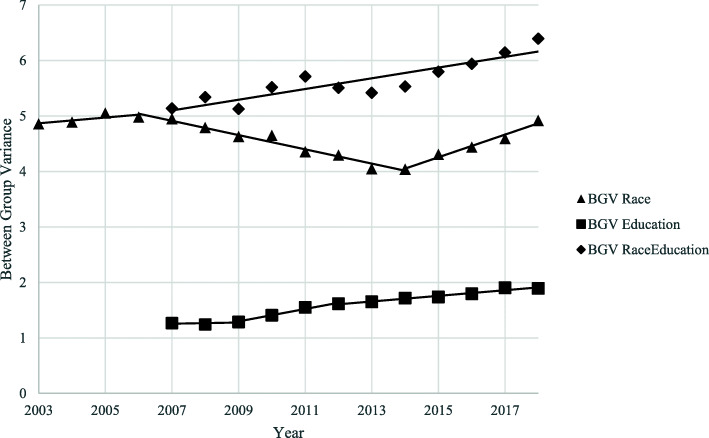
Trends of Disparities in LBW by Maternal Race, Education, and Their Combination, United States, 2003–2018. LBW = Low Birthweight. BGV = Between Group Variance

BGV trends are shown in Fig. 5. The racial disparity was substantially larger than the educational disparity, by between 2 and 4 times as much. From 2003 to 2006, the racial disparity increased with an APC of 1.4, significantly decreased from 2006 to 2014 with an APC of − 2.8, and significantly increased again from 2014 to 2018 with an APC of 4.9. The educational disparity increased over the entire time period, with an APC of 1.6 from 2007 to 2009, a significant APC of 8.0 from 2009 to 2012, and a significant APC of 2.7 from 2012 to 2018. The BGV for the 20 distinct, race by education subgroups also significantly increased between 2007 and 2018 at a rate of 1.7% per year, driven largely by the racial disparities.

When these analyses were restricted to percentage of VLBW, similar patterns of trends were observed with the exception of slightly decreasing racial disparities, potentially due to decreasing percentage of VLBW in more highly educated Black mothers (see Additional file 1). Similar patterns of findings also applied to mothers of advanced maternal age, though percentage of LBW experienced less decline in the middle time period. Finally, trends in percentage of LBW for multiple births had comparable results as well, barring less increase in the most recent time period and more strongly increasing disparities. Ultimately, these results suggest that trends and disparities observed for percentage of LBW in the overall population may be comparable across these maternal and infant subgroups.

## Discussion

Results from this study showed that, despite some improvements in percentage of LBW from 2006 to 2014, recent trends are troubling. Since 2014, the trends in percentage of LBW have experienced a significant turn for the worse, negating nearly a decade of steady improvement in the rates. This reversal or worsening was detected for the overall population of the U.S., as well as for nearly every subgroup explored. In fact, for every maternal race by education subgroup except for AIAN mothers with a college degree or higher, LBW rates in 2018 exceeded those at the start of the period in 2007. The negative trend that began around 2014 was particularly stark for mothers in populations of color with lower education.

Also troubling is the increasing disparities observed over this time period. Since 2014, racial, educational, and race by education disparities all increased. So, not only has the magnitude of percentage of LBW been on the rise since 2014, so too have the differences between subgroups. Particularly stark is the disparity between White mothers with high education and Black mothers with low education, with the latter experiencing LBW rates over three times the former. Our findings clearly confirm that the advantaged groups and disadvantaged groups in the context of our nation’s social hierarchy are only becoming more so. The lack of continued progress on percentage of LBW and the increasing racial and socioeconomic gap across the U.S. may be due to any number of contextual factors. The particular timing of the change in trajectory is specifically intriguing. Some researchers have posited that the macroeconomic downturn of the Great Recession (2007–2009) may play a role in this worsening trend and increasing racial disparities [22–24]. For instance, similar findings have shown a worsening trend in life expectancy and cause-specific mortality, including disparities in race and education, in the U.S. since at least 2014 [25, 26]. Similarly, others have explored trends in birth outcomes as they relate to access to prenatal care and smoking behavior within the context of macroeconomic improvements [27]. Though difficult to account for macroeconomic indicators while controlling for individual-level covariates, this growing body of evidence provides one potential explanation for recent worsening trends and growing disparities. This question of what may be causing declines in length and quality of life is a promising area for continued research. Further worth noting is the fact that racial disparities in percentage of LBW were much greater than educational disparities, and, when considered together, educational attainment could not explain away racial disparities. This implicates additional factors such as structural and interpersonal racism, stress, and other socioeconomic risk factors that disproportionately burden mothers in populations of color, particularly those who are Black. For example, mechanisms contributing to heightened inequities for Black mothers as noted in the literature, among many others, include unequal distribution of social resources; living in disadvantaged neighborhoods due to residential segregation; racial discrimination and chronic stress, which limit opportunity, inflict emotional and physical harm, and result in physiological wear and tear; and differential allocation of health care, such as receiving less medical advice, information about risks and complications, and common prenatal treatment [9, 28–33].

While the focus of this study was on the relative disparities between race, education, and race by education subgroups, differences in the absolute occurrences of infants born at LBW are also important to consider as they represent the burden on the population. As an example, data showed that despite having less than half the LBW rate of Black mothers (5.3% vs 11.7%), White mothers accounted for close to twice as many absolute infants born at LBW (101,000 vs 65,000), many more of which were to the highest educated Whites as compared to the lowest educated Whites (39,000 vs 12,000). The wide-ranging consequences of LBW that burden the social service, education, and health care systems – from infant mortality to growth and cognitive problems in early life, to hypertension, diabetes, and cardiovascular disease later in the child’s life – highlight the fact that both rate and burden are valuable considerations for population health outcomes, and each deserve research and policy attention [3–7, 34].

There are several important things to note when reflecting on the findings of this study. For instance, due to changes in data collection, fewer years of study were able to be analyzed for the education subgroups. Furthermore, as an outcome measure, LBW has its limitations. This outcome measure was chosen for this study because of its reflection of infant and maternal health, its understandability to policy makers, and the quality and availability of its data at many levels. However, the defined cut-off for LBW at < 2500 g is somewhat arbitrary. Researchers have also called attention to assumptions widely held by the public health community about LBW, such as its utility as a marker of population health and, specifically, infant mortality risk. This study did not explore trends in other related measures of birth outcomes, such as preterm birth, small for gestational age, or infant mortality, which themselves have analytic limitations but could offer a refined understanding of and specific focus on perinatal risk [3].

Additionally, disaggregating birthweight data by subgroups such as race/ethnicity or education groups can pose some issues. For example, babies may have differing birthweight distributions according to the ethnic origin of the mother, such as babies born to Asian mothers who tend to be smaller, which may misclassify them as at risk of increased morbidity [35, 36]. The race/ethnicity and education subgroups also reflect the race/ethnicity and education of the mother, not the child itself. With many babies being born to parents of different races and different educational backgrounds, this classification may miss key aspects of the child’s underlying characteristics and risk. In using mutually exclusive racial/ethnic categories, distinctions between babies born to mothers of multiple race/ethnicity groups were also not captured. The broad definition of Hispanic includes Hispanics of different country origins and races, such as Black-Hispanics and American Indian-Hispanics, which have historically very different experiences in the U.S., and thus differences in these subgroups were not portrayed. Our method of analysis to determine disparities and to detect trends also did not allow for more advanced adjustment techniques to account for covariates, such as important demographic or behavioral factors, that may vary by group and affect percentage of LBW.

Finally, it is possible that the worsening trends in percentage of LBW detected in the current study could be due, in part, to additional, extraneous reasons that are less critical from a population health perspective. For example, increases in multiple births (in part due to the increased use of in vitro fertilization), women choosing to have children later in life, greater insurance coverage of infertility treatment, and improvements in technologies used to monitor and improve outcomes for at-risk pregnancies are all associated with smaller infants [20, 21, 37–40]. However, while these factors may play a role in the worsening percentage of LBW trends, secondary analyses of advanced maternal age and multiple births did not find strong discrepancies in these factors as compared to overall percentage of LBW. Furthermore, delayed child bearing and the utilization of advanced medical technology, even when costs are lowered and access is increased, is more often among mothers of higher socioeconomic status, yet this study found larger increases in percentage of LBW among mothers of low education [39–41]. Therefore, it is unlikely that these factors are driving the worsening trends in percentage of LBW.

Continued monitoring of recent trends is needed to see if they are temporary or representative of a persisting reversal of the progress made in reducing percentage of LBW over more than a decade. It is also important for future studies to analyze these trends and disparities at a finer grain geography, such as the state level, to understand whether these trends hold true across all states, or manifest in particular states or regions. Supplementary research could also be valuable in further exploring the under-studied population of multiple birth infants and how types of fertility treatment may impact these findings, with newly available information offered in CDC WONDER’s Natality Files starting with the year 2016. Furthermore, initiating some target-setting practices would be recommended to understand and communicate what we need to do as a country in order to embark on a path towards better health for our mothers and infants. It should be noted that the three different trend components in Fig. 1 make the initial purpose of this analysis for aiding in future target projections complex and underline the importance of time period selection for such trends. Using the overall trend line would support stable future targets, but this is contradicted by the trend of both the early and late time components. Finally, and most importantly, measuring racial disparities in outcomes will only get us so far. We acknowledge the need to move our study and inquiry upstream to capture the racism and power imbalances at the root of these disparities and to establish that they represent societal inequities. While this is no small feat, it is required as the field takes steps towards health equity research.

## Conclusion

The percentage of infants born at low birthweight (LBW) as a measure effectually reflects the population health experience of society, representing maternal health and both current and future child health. Therefore, understanding current levels, disparities within the population, as well as past and future trends of percentage of LBW in the United States is imperative to improving health for all in our nation. This study showed that trends in percentage of LBW significantly worsened in the 5–6 years leading up to 2018. This recent rise directly nullifies the improvement our nation experienced in the previous decade. Race and class disparities in percentage of LBW have also been on the rise in recent years, further separating the divide between advantaged and disadvantaged groups. These increasing disparities are directly in conflict with achieving population health and equity in the U.S. Balancing considerations of both rate and burden for LBW is also essential for research and policy. Finally, this study has implications for future research. We must continue to monitor these trends, understand how they are playing out across the country, incorporate measurement of upstream drivers, and set targets for our nation in order to effectively make progress towards equitable birth outcomes and maternal health.

## Supplementary Information

**Additional file 1.** Data and Trends for LBW, VLBW, Advanced Maternal Age, and Multiple Births by Maternal Race, Education, and Their Combination, United States, 2003–2018. An additional file is provided, entitled ‘Additional File 1.xlsx’, containing the datasets supporting the conclusions of the article. The first sheet entitled ‘LBW Data’ includes the number of infants born at low birthweight, the total number of births, and the percentage of infants born at low birthweight overall, by race/ethnicity, by education, and by race and education subgroups for the years under study. The second sheet entitled ‘VLBW Data’ includes the same information for infants born at very low birthweight. The third sheet entitled ‘VLBW Trends’ includes the trend period years and annual percent changes for all groups and between group variances for infants born at very low birthweight. The fourth sheet entitled ‘AMA Data’ includes the number of infants born at low birthweight, the total number of births, and the percentage of infants born at low birthweight overall, by race/ethnicity, by education, and by race and education subgroups for mothers greater than or equal to 35 years of age and for mothers less than 35 years of age. The fifth sheet entitled ‘AMA Trends’ includes the trend period years and annual percent changes for all groups and between group variances for infants born at low birthweight for mothers ages 35 and over and for mothers less than age 35. The sixth sheet entitled ‘Multiples Data’ includes the number of infants born at low birthweight, the total number of births, and the percentage of infants born at low birthweight overall, by race/ethnicity, by education, and by race and education subgroups for multiple birth infants. Finally, the seventh sheet entitled ‘Multiples Trends’ includes the trend period years and annual percent changes for all groups and between group variances for multiple birth infants born at low birthweight.

## Data Availability

The datasets supporting the conclusions of this article are available in the CDC WONDER repository, https://wonder.cdc.gov/natality.html. The datasets are also included within the article (and its additional file).

## Abbreviations

LBW: Percentage of infants born at low birthweight
AIAN: American Indian and Alaska Native
APC: Annual percentage change
BGV: Between group variance
VLBW: Percentage of infants born at very low birthweight
